# Phytochemicals and pharmacology of pomegranate (*Punica granatum* L.): nutraceutical benefits and industrial applications: a review

**DOI:** 10.3389/fnut.2025.1528897

**Published:** 2025-03-27

**Authors:** Sana Noreen, Bushra Hashmi, Patrick Maduabuchi Aja, Ayomide Victor Atoki

**Affiliations:** ^1^University Institute of Diet and Nutritional Sciences, The University of Lahore, Lahore, Pakistan; ^2^Faculty of Biomedical Sciences, Kampala International University, Western Campus, Bushenyi, Uganda

**Keywords:** pomegranate, anti-inflammatory, anti-oxidant, anti-microbial, anti-fungal, anti-obesity, anti-diabetic, hepatoprotective activity

## Abstract

**Introduction:**

Pomegranate (*Punica granatum* L.) is a fruit native to South Asia and currently can grow in tropical and subtropical areas, which produces approximately seven thousand metric tons per year. Pomegranate stands out for its rich flavor and functional properties, which is why it has gained acceptance in different countries. Beyond its health advantages, it has industrial applications in food technology, cosmetics, and medicines. This study focuses on its diverse phytochemical profile and the medicinal properties of its bioactive components.

**Methods:**

A search in PubMed, Scopus, EBSCO, Medline, PubMed, Embase, SID, and Iran Medex databases was conducted to identify clinical and observational studies on Pomegranate consumption and its industrial uses.

**Results:**

Pomegranate and its by-products are rich in beneficial phytochemicals, provide health benefits, and help manage ailments. Sustainable reuse of its by-products supports health, economic growth, and food security.

**Conclusion:**

Pomegranate provide health benefits, including antidiabetic, antioxidant, anticancer, and anti-inflammatory effects, with potential for food product development and disease management. Analyzing pomegranate's functional and nutritional properties, especially its peel and seed, is crucial for understanding the mechanisms involved in industrial processes for nutraceutical or functional food products.

## Introduction

All fruits and vegetables are linked which health health-enhancing activity as they are richly endowed with essential nutritional components. Various investigations about the uses of medicinal plants identified that not only fruits and vegetables but their other parts such leaf, peels, and seeds are also possess beneficial activities (1). Pomegranate (*Punica granatum* L.) is also a medicinal fruit that is traditionally used throughout the world. The cultivation of pomegranate is prevalent in Mediterranean regions, including Asia, particularly in Pakistan, India, and Iran. Pomegranate is considered as one of the first cultivated fruit and is known for it's a beneficial effect on human health. Over the past 4,000 years, pomegranate has primarily been cultivated for its medicinal activities and health-improving effects (2, 3). Studies conducted in the twenty-first century have reported that pomegranate is rich has nutritional value and possesses extensive therapeutic activities properties. Due to the growing demand for its consumption, pomegranate production has increased to meet this demand (4). Various parts of the pomegranate fruit, including as the seeds, root, leaves and peel, contain multiple bioactive compounds and their the mechanism of action has the potential to treat multiple pathophysiological conditions (5).

**Figure 1:**
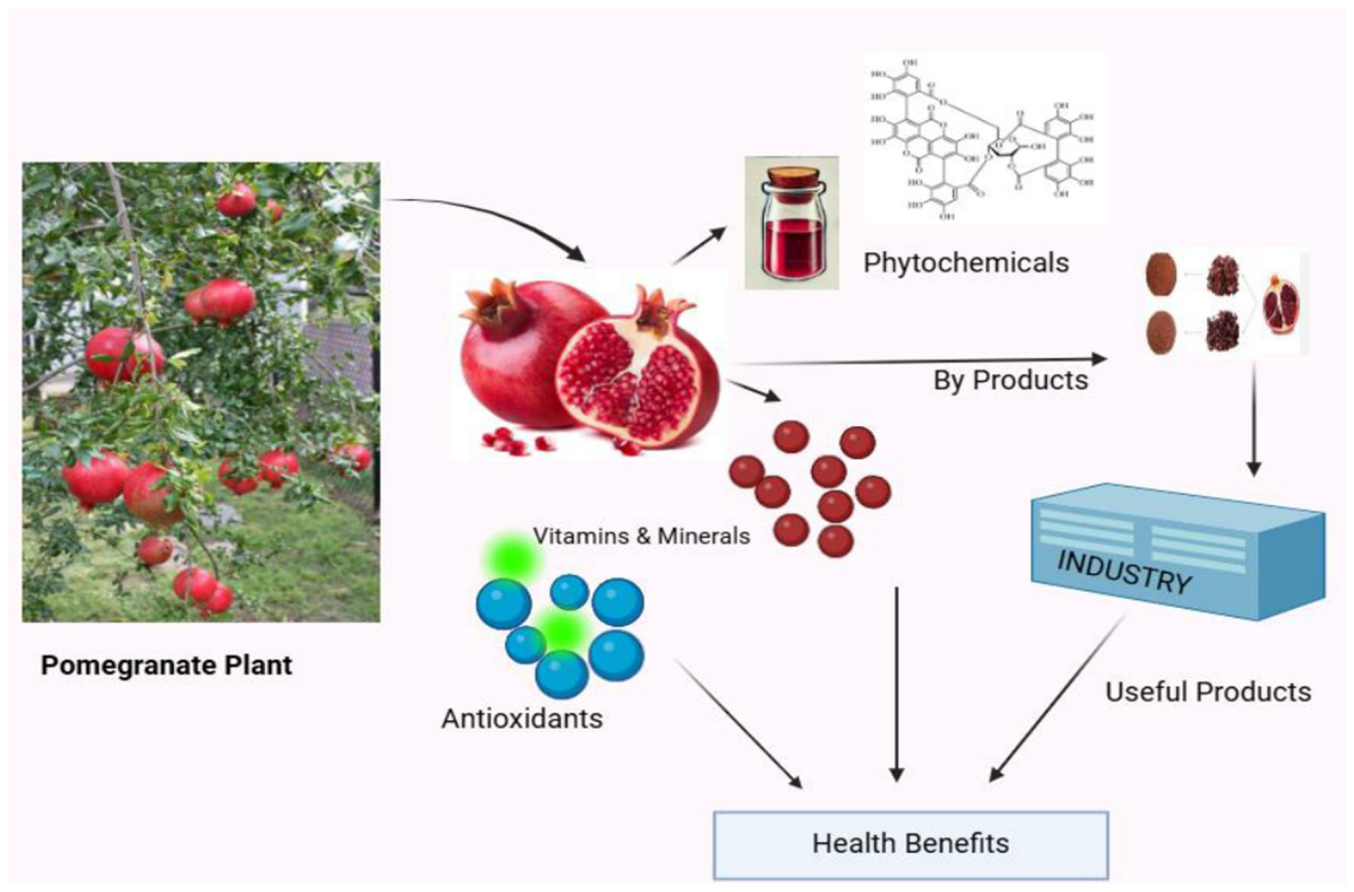
Health benefits of pomegranate (*Punica granatum* L.).

A wide range of phytochemicals, polysaccharides, vitamins, minerals, and carbohydrates are present in high amounts in various parts of pomegranate fruit such as the leaf, seed and root. The seeds of pomegranate contain alpha-linolenic acid, linoleic acid, oleic acid, and fatty acids that have many nutraceutical properties as mentioned in Figure 1. The mechanism of action of different phenolic components present in different parts of pomegranate is shown in multitudinous studies. These components exhibit potent antibacterial, antioxidant, anti-inflammatory, antidiarrheal, antidiabetic, antiviral, and anti-obesity properties, help to protect from many other health-related issues. Due to the immense potential of bioactive and phenolic content in pomegranate peel, seed, and leaf, it has emerged as a powerful traditional medicine for treating human pathological conditions. Various functional foods made from pomegranate byproducts, that contain high concentrations of bioactive compounds, have been developed (6). Clinical trials have shown significant results combating chronic diseases due to their therapeutic effects. These bioactive compounds are also and beneficial for many traditional uses, such as prevent cancer, diabetes, heart problems, and many other diseases (7, 8).

**Figure 2:**
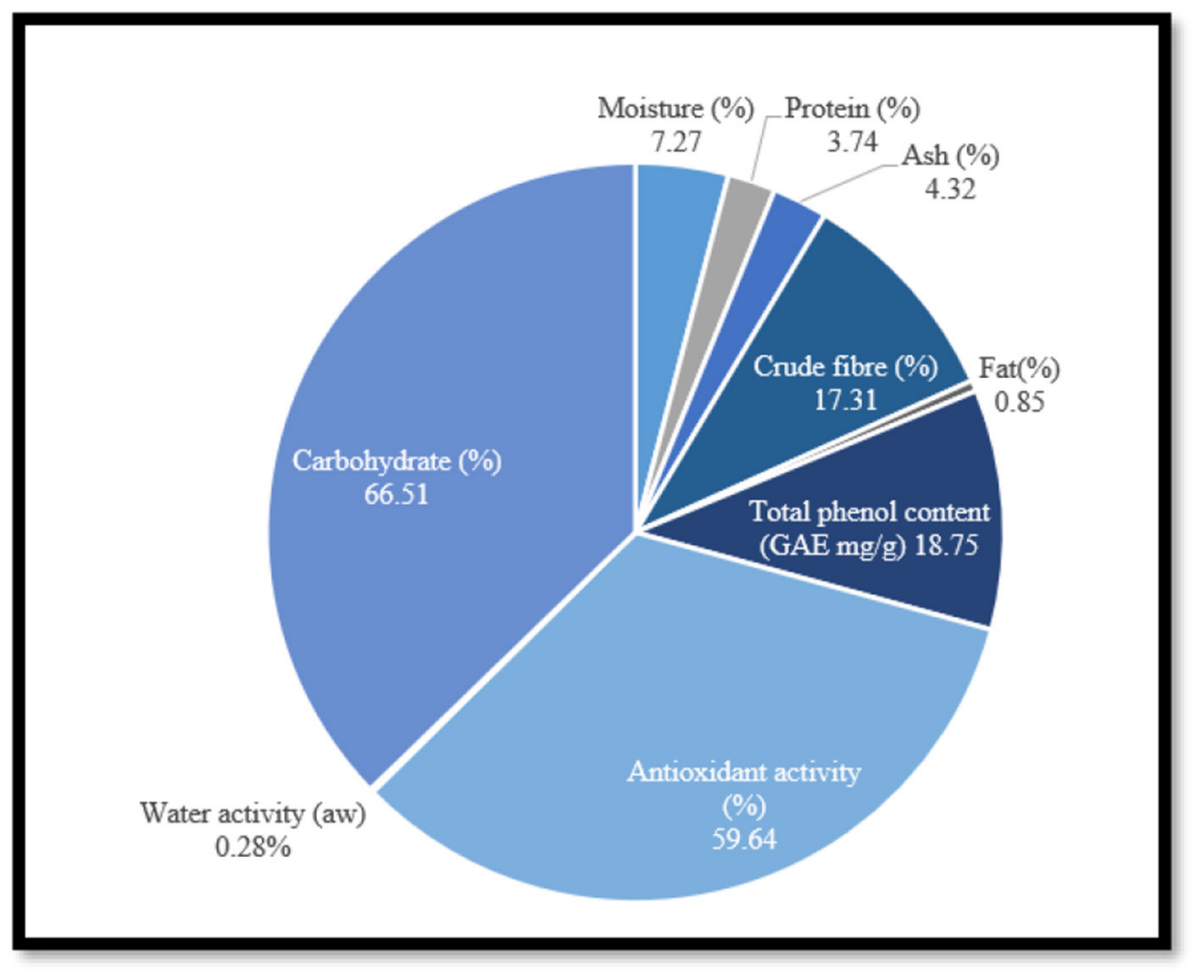
The chemical composition of fresh pomegranate peel powder.

The therapeutic efficacy of fruits and their various parts due to their valuable chemical compounds make them highly beneficial. Pomegranates contain a higher amount of nutrients compared to many other fruit plants, providing giving 80 kcal per 100 g of serving. The inedible portions, such as peel and seeds, are also good sources of nutrients, though they are often wasted are by food industries. Byproducts of pomegranate juice such as peel, seed, leaf, and husk comprises high quantity of nutraceutical properties. Pomegranate peel, which comprises 30–40% of fruit, offers a variety of offers beneficial biological effects and could help resist pathological problems. The loss of waste products, such as inedible portions of fruits, not only increases food waste but also contributes to loss of essential nutrients (9). Waste products of fruits and vegetables are abundant with substantial quantities of polyphenols. Among, these byproduct, the peel of pomegranate is particularly enriched enriched abundant in major bioactive compounds, including flavonoids, tannins, and other phenolic compounds. Pomegranate peel comprises 26–30% of the total weight of fruit which contains several bioactive compounds such as anthocyanins, catechins, punicalagin, and ellagic acid. The chemical composition of fresh pomegranate peel powder is presented (10) in Figure 2. This study focuses on its diverse phytochemical profile and the medicinal properties of its bioactive components.

## Method

To gather the relevant material, the keywords “pomegranate”, “waste product”, “therapeutics”, and “pharmacology” were searched in journals accessible through databases such as ScienceDirect, Scopus, EBSCO, Medline, PubMed, Embase, SID, and Iran Medex. Duplicate papers were removed, and only one version of each was retained.

### Phytochemical composition of pomegranate

**Figure 3:**
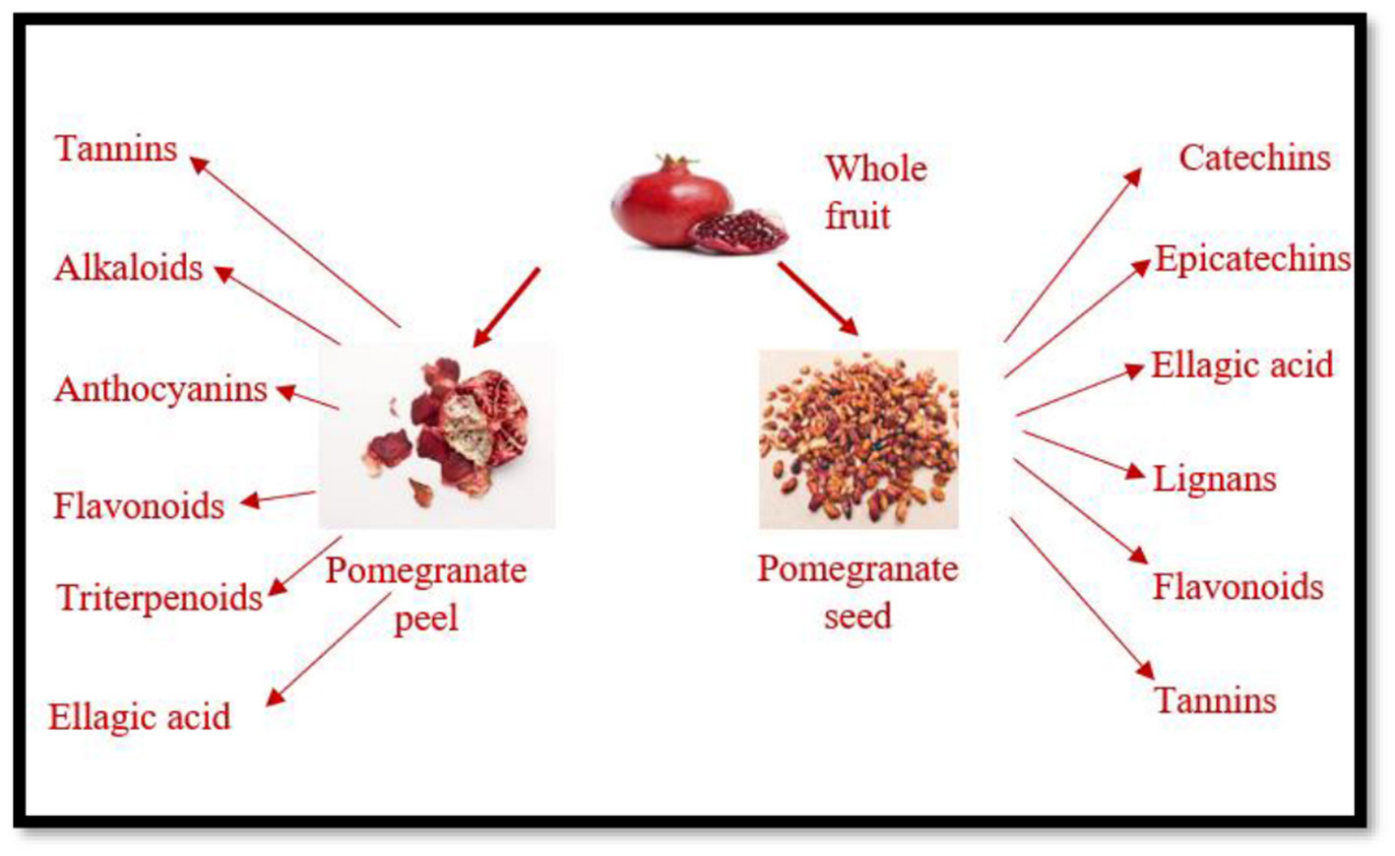
Phytochemical and botanical description of pomegranate peel and seeds.

The experimental results of many studies have shown that the bioactive compounds in the extract of pomegranate peel leaf, and seeds the possess curative properties and with the potential to treat various health-related pathological conditions. Among all the bioactive compounds in pomegranate peel, tannins, flavonoids, alkaloids, phenols, flavonoids, alkaloids, steroids are present in abundance. In addition to phenolic compounds, pomegranate peel also contains various dietary fiber, vitamins, and minerals. These compounds have the potential to support and maintain physiological functions. The phytochemical constituents are present in pomegranate and its parts make them useful for treating different human ailments were, the correlation between these phytochemicals and human ailments showed a strong potential connection, results showed showing significant improvement in human health (11). In general, the bioactive composition of pomegranate peel showed a higher concentration of phenolic content. The pomegranate peel extract demonstrated a significant antioxidant, antifungal, and antibacterial effect due to the presence of phenolic phytochemicals (12, 13). The multi-functionality and health-promoting effects of pomegranate seed and peel as demonstrated in Figure 3.

### Health benefits of pomegranate

**Figure 4:**
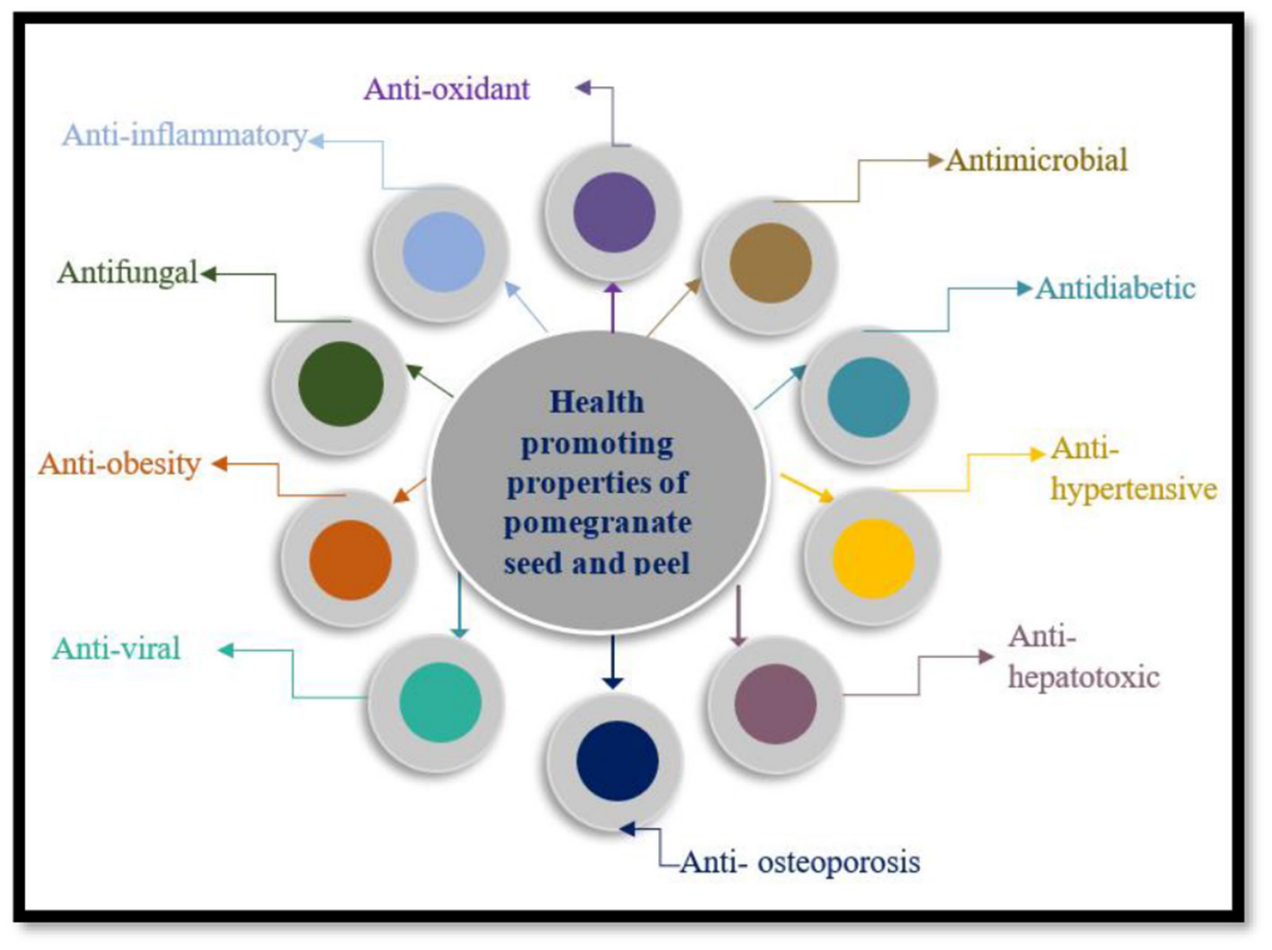
Health benefits of pomegranate peel and seed.

Pomegranate and its various parts are nutritionally rich and provide exceptional benefits to overall human health. Pomegranate contains many bioactive constituents that play a major role as functional foods and contribute to the improvement of human health. The most predominant phytochemical in pomegranate are anthocyanins and hydrolysable ellagitannins, which primarily contribute to the inhibition of cancer cell and help to eliminate free radicals (14). Several studies have brought up the potential of many bioactive compounds like polyphenols that the can treat and manage metabolic diseases like diabetes, and heart issues. Many studies have shown promising results regarding pomegranate peel against Alzheimer's disease it's ability to improve cognitive behavior. Several others studies have discussed how pomegranate peel contributes in protecting against liver injury. All bioactive compounds in pomegranate and its inedible portion possess varieties of phenolic compounds that exert a positive impact in treatment of various chronic diseases (15). Furthermore, pomegranate and it constitutes have health promoting properties such as antihypertensive, antidiabetic, anticancer, antibacterial, antifungal, anti-obesity, and anti-inflammatory as shown in Figure 4.

### Antioxidant activity of pomegranate

Several studies have demonstrated that among different parts of pomegranate, the peel enriches with the highest amount of total polyphenolic compounds, flavonoids, carotenoids, and alkaloids. The inedible portion of fruits contains more beneficial components, which help reduce the risk of several chronic degenerative diseases. The phenolic composition of pomegranate peel has shown significant results in combating oxidative stress (16). Several studies have highlighted the beneficial role of polyphenols present in pomegranate extract. The major phenolic constituents, such as ellagitannins, contribute significantly to its potential antioxidant activity. The results have shown that pomegranate extract is an excellent source of the key antioxidant components. Additionally, the extract of isolated pomegranate seeds when treated with different solvents like acetone, ethyl acetate, and methanol, examine was evaluated to examine the antioxidant properties of pomegranate seeds. The antioxidant activity of methanol extracts of pomegranate seeds showed the highest antioxidant capacity compared to other solvents proving that the phenolic compounds possess strong radicals scavenging properties. The results showed that the methanol extract of pomegranate seeds has a greater ability to reduce oxidative stress. Several studies have also shown that the leaf extract of pomegranate contained a high proportion of phenolic compounds, including hydrolyzable tannins, punicalagin, anthocyanins, and ellagic acid. The beneficial effects of antioxidants on human health lie in their ability to remove free radicals from the human body. Therefore, promoting the use of these anti-oxidant-rich components in food products can significantly contribute to helping improve human health (17). An aqueous suspension of pomegranate peel powder was examined to test it's antioxidant capacity, and the result showed that the peel has strong antioxidant properties. Phenolic compounds like tannins and flavonoids are the major components in pomegranate peel, which are associated with the defensive mechanism against deleterious effect of free radicals and contribute to reduce oxidative stress. Therefore, the antioxidant potential of aqueous suspension of pomegranate peel powder has the potential to protect the human body from chronic disease like cancer (14). The hydrolysable tannins and punicalagin in pomegranate peel exhibit high antioxidant capabilities, acting as reducing agents against reactive oxygen species (18). The pomegranate seed oil (PSO) showed reduction in lipid oxidation and inhibit the progression of oxidation. High radical scavenging activity of punicic acid, which is major residue in PSO, contribute in protection from free radicals. The PSO exhibited potential antioxidant activity by inhibiting lipid oxidation (19).

Pomegranate seed oil, rich in polyphenolic content and punicalagin, has been found to improve skin health, inhibit inflammation, and aid in cosmetic product production (20).

### Anti-inflammatory activity of pomegranate

From centuries, the pomegranate used to treat inflammation due to its potential anti-inflammatory capacity. Pomegranate (*Punica granatum* L.) leaf (PGL) has many health beneficial activities including anti-inflammatory properties and neurodegenerative properties. This study investigated that pomegranate extract possesses strong anti-inflammatory property against inflammation as the extract showed rich content of ellagitannins (21). Recent few studies identified those constituents of pomegranate such as ellagic acid (EA) has promising pharmacological effects. EA is majorly active compound in pomegranate that possesses strong action mechanism against inflammation. Several studies suggest that EA also involve in other pharmacological effects such anticancer, antiaging and anti-mutaganic activities (22). Studies showed that pomegranate peel extract has anti-inflammatory properties, reducing pro-inflammatory activity and regulating inflammatory markers, thereby improving liver enzyme activity and reducing liver inflammation (23). This study investigated the inflammatory enzyme cyclooxygenase (COX-2) that involve in process of inflammation. The ethanol extract of pomegranate peel showed inhibitory effect against inflammatory activity of COX-2 enzyme by suppressing the synthesis of prostaglandins, which are pro-inflammatory mediators. The anti-inflammatory action mechanism ethanolic extract of pomegranate peel showed significant results against pro-inflammatory cytokines and COX-2 enzyme (20).

**Figure 5:**
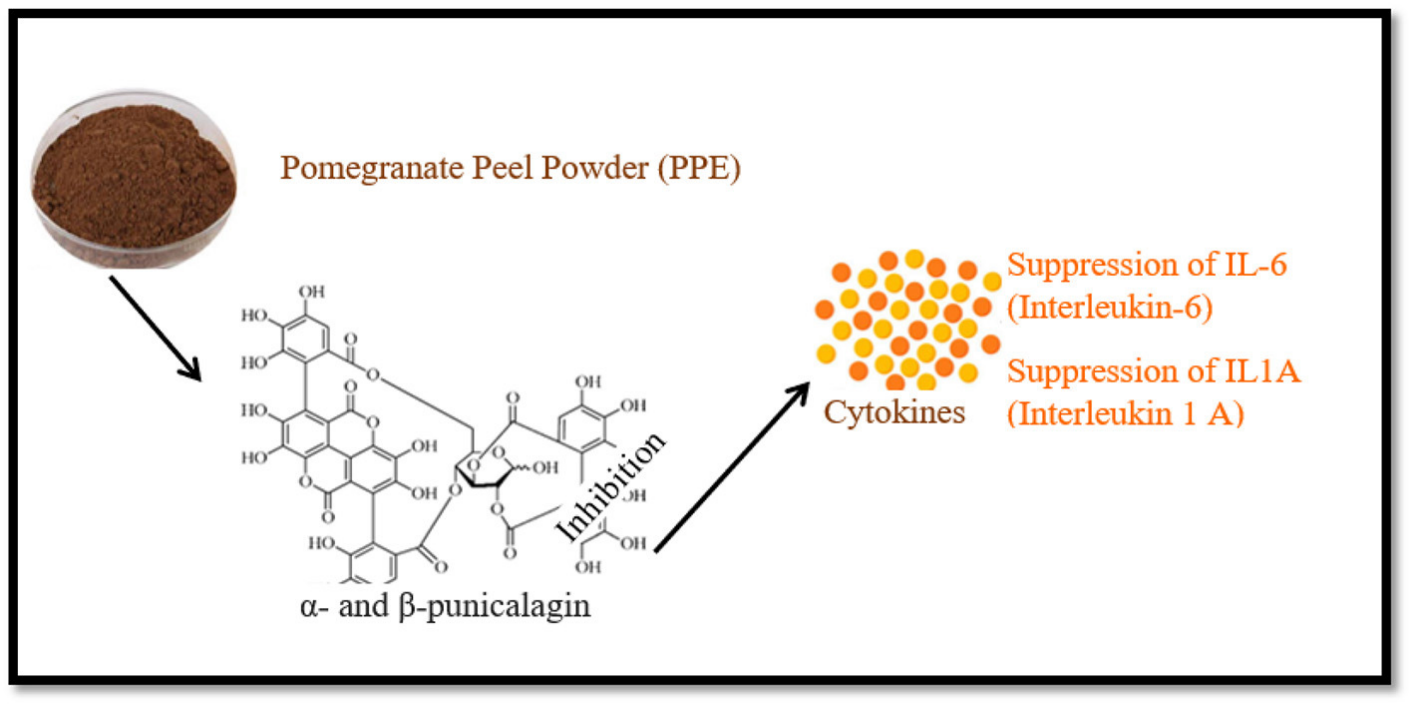
The inhibited effect of Pomegranate peel powder, α- and β-punicalagin on pro-inflammatory cytokines IL6, and IL1A.

Pomegranate husk exhibits anti-inflammatory properties due to phenolic components like ellagic acid, suppressing nitric oxide and pro-inflammatory cytokines. Recent studies show its peel extract has anticancer, antihypertensive, and anti-hypoglycemic effects (20). PPE significantly showed anti-inflammatory capacity and other impressive therapeutic applications. The α- and β-punicalagin from the PPE showed a significant inhibited effect against pro-inflammatory cytokines (24) as mentioned in Figure 5.

### Anti-microbial activity of pomegranate

Pomegranate leaf, peel and seed have high number of bioactive compounds. The importance of pomegranate peel is more than other parts of fruit as it is major by-product during food processing. High potential of pomegranate peel is beneficial in preservation process as it helps to prevent food from deterioration. Pomegranate peel extract (PPE) considered as natural additive and contribute for better of quality of food. PPE is abundant with natural compounds that contribute in bio functional edible film for packaging of food products (25, 26). Number of phenolic compounds in pomegranate peel and seed have strong antimicrobial property against foodborne pathogens. Recent study showed pomegranate peel extracts with concentration (0.33 g/mL) possesses inhibited effect against *Staphylococcus aureus. Staphylococcus aureus, Bacillus cereus, coli, Pseudomonas aeruginosa* (13). The high level of bioactive compounds in the 2 g pomegranate peel extract with 10 mL of methanol tested to identify antimicrobial activity. Total phenolic compounds, total flavonoids, and tannins have potential antimicrobial activity against *Escherichia coli, Klebsiella pneumonia, Bacillus subtilis*, and *Staphylococcus aureus*. The bioactive compounds of pomegranate peel extract have biological constituents, which ensure maximum antimicrobial effect against growth of microbes (27). This study evaluated the antimicrobial effect of pomegranate peel and seed with different extracts including ethanol and methanol against five pathogens: *Bacillus* sp., *Staphylococcus aureus, Pseudomonas* sp., *Escherichia coli*, and *Aeromonas hydrophila*. The ethanolic extract of pomegranate peel showed maximum and significant inhibition at concentration of 15 mg/mL against all five pathogens than methanol extract. The antimicrobial activity of components founded in pomegranate peel and seed extract provided resistance against pathogens by damaging cytoplasmic membrane of microbes and deleting their genetic bands (28, 29) (Table 1).

**Table 1:** Anti-microbial effect of different pomegranate extracts against various microbes.

**Microbes**	**Dose of sample and solvents**	**Action of mechanism**	**References**
*Bacillus cereus, Bacillus subtilis, Enterococcus faecalis*	10 g pomegranate peel extracted with 100 mL of solvents (ethanol, methanol) combination	Due to high content of punicalagins and ellagic acid, significant antimicrobial activity was observed (*P* < 0.05).	(12, 29)
*E. coli*	1 g pomegranate peel and 100 mL of DI water	Effect of tannins and punicalagin, exhibit potential antimicrobial activity on bacterial membrane.	(8)
Streptococcus mutans *R. dentocariosa*	5 g peel hydroalcoholic extracts and 25 mL of ethanol	Inhibited effect of anthocyanins, hydrolysable tannins punicalagin and punicalin on growth of microbes.	(49)
*Bacillus* cereus, *Pseudomonas Aeruginosa, B. cereus* *Staphylococcus aureus*	8 g Pomegranate peel and 500 mL of water	Total phenolic content showed significant antimicrobial activity.	(31)

### Anti-fungal activity of pomegranate

**Figure 6:**
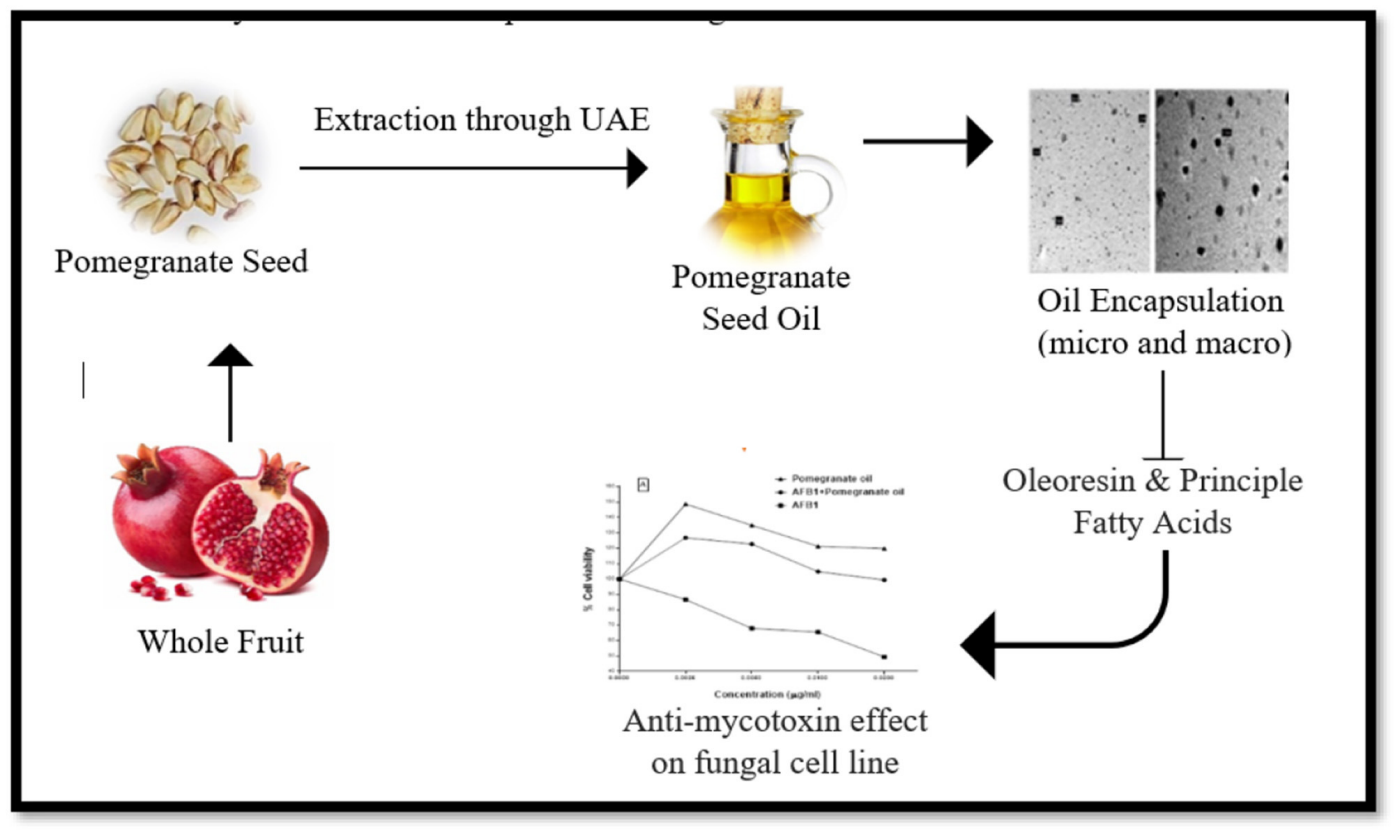
Impact of pomegranate seed oil, oleoresin and principle fatty acids on formation of mycotoxin.

Most of pomegranate peel and seeds are considered as waste but these inedible parts have substantial antimicrobial activities. Pomegranate peel and seeds are served as important potential source of antifungal agents. The seed and peel extracts of pomegranate have antimicrobial and antifungal activity against various human ailment. Anthocyanins, tannins and ellagic acid showed antifungal mechanism of action by destroying the cell membrane of pathogens and disturb their growth activity. The study investigated that the aqueous extract of pomegranate peel and seed possesses strong inhibited effect against mycelial growth of *Aspergillus niger* (30). The polyphenolic aqueous extracts of pomegranate peel have maximum content of polyphenolic compounds that possesses antifungal activity. These phenolic compounds inhibit proliferation of fungal enzymes and cause disruption of fungal cell growth and results in reduction of fungal spread and reproduction (31, 32). Recent study discussed that pomegranate peel powder has highest number of phenolic compounds particularly Ellagic acid (EA). These components showed significant antifungal effect against the growth of Aspergillus fumigatus and Candida spp. The findings of study represented that (EA) showed effective antifungal mechanism by disturbing fungal cell wall, protein membrane and results showed death of fungal cell. The minimal fungicidal concentrations (MFC) of pomegranate powder (100 μg/mL) showed significant inhibition process against *Candida albicans* (33). Edible oils have significant level of monounsaturated fatty acids and these oils are also majorly use in food industries as a food ingredient. Pomegranate seed have enough source of oil and possesses potential benefits in food industries as by product. Pomegranate seed oil significantly showed potential antifungal activity against various types of fungus including *Fusarium oxysporum* f. sp. lycopersici, *Botrytis cinerea, Sclerotinia sclerotiorum, Botrytis cinerea*, and *Rhizoctonia solani*. Several studies determined the inhibited effect of pomegranate seed oil against various fungus growth (34). This study demonstrated that methanol extract of pomegranate peel and powder contain major antifungal phytocomponents such as sterols, flavonoids and glycosides. These components exert potential antifungal effect against phytopathogenic fungi including *Rhizoctonia solani, Botrytis cinerea, Phoma* sp., and *Colletotrichum dematium* by inhibiting their growth and spore germination in host cell (35). This study investigated the antifungal effect of methanolic extract of pomegranate peel against *Fusarium sambucinum*. The phytochemical components specifically in punicalagin, epicatechin, tannins, punicalin, and flavonoids in methanolic extract of pomegranate peel showed significant inhibitory effect by suppressing the mycelial growth at a maximum concentration. These components exhibited potential inhibition on spore germination of fungus, inhibit their further progression, and thus prevent from fungal toxicity (36). The pomegranate seed oil rich bioactive phytochemicals including carotenoids, tocochromanols, sterols, phenolic, and flavonoids, which have ability to reduce the effect of mycotoxin formation presented in Figure 6.

### Anti-obesity activity of pomegranate

Globally, obesity has become major initiator to contribute to progression of several metabolic disorders. Results of several studies showed that pomegranate seeds oil (PSO) play central role in many pharmacological problems. PSO contain many important phytochemicals, one of them is Punicic acid (PA) that possesses potential anti-obesity effect and effective against various metabolic disorders. PSO have ability to treat obesity by suppressing intestinal glucose absorption, improving lipid profile and controlling body weight (37). The results of previous study showed that the diverse phytochemicals of aqueous and ethanolic peel extracts of pomegranate have inhibitory properties against pancreatic lipase. The ethanolic extra contain phenolic acid, glycosides, tannins, flavonoids, and other components. Due to the presence of these phytochemical components, ethanolic extract showed more significant results against lipase with value of 603.50 μg/mL (38). Lipase and α-amylase are two enzymes that are important in obesity management. The multifunctional component present in pomegranate peel including tannins, flavonoids, and phenolic acids have ability to inhibit the enzymes activity which are involve in progression of obesity. The phytochemical components immobilized the activity of lipase and α-amylase and help in the treatment of obesity (39). The Chemical composition of PA has the capacity to decrease the lipid accumulation and deposition of fatty acids. PSO has great potential in development of various nutraceuticals foods that use to treat obesity and its related other metabolic issues. Despite this, PSO also acquires ability to modulate the inflammatory biomarkers. The phytochemical profile of PSO make it more useful in development of functional foods (40). The results of study explored that pomegranate peel has ability to reduce body weight by modulating the composition and activity of gut microbiota. The pomegranate peel showed significant results against obesity by improving glucose and lipid metabolism. The finding of study suggested that correlation between phytochemical components of pomegranate peel and obesity-related biomarkers were significant (41). The peel extract of pomegranate has an ability to manage body weight by reducing subcutaneous adipose tissue and appetite. Thus, help to improve lipid profile to reduce weight. The results of study revealed that combination of pomegranate peel extract and probiotics exert significant effect to prevent and treat obesity. The polyphenolic content in pomegranate peel specifically ellagic acid, anthocyanins, punicalagins, and ellagitannins, which play important role in treatment of obesity. These components and probiotics treat the obesity by lowering triglycerides level, lipid accumulation and inhibited the process of adipogenesis in cell. The anti-obesity mechanism of phytochemical content contributes in prevention of obesity (42). The results of the study showed that the supplementation of pomegranate peel extract exerts significant effect against obesity and osteoarthritis in obese women. A wide range of phytochemical components exhibits potential anti-obesity effect by mechanism action of organic acids, anthocyanins, punicalin, alkaloids and other beneficial properties on progression of obesity. The supplementation potentially involves the reduction of dyslipidemia, cholesterol, fat cells (adipocyte) and body mass (43).

### Anti-viral activity of pomegranate

**Figure 7:**
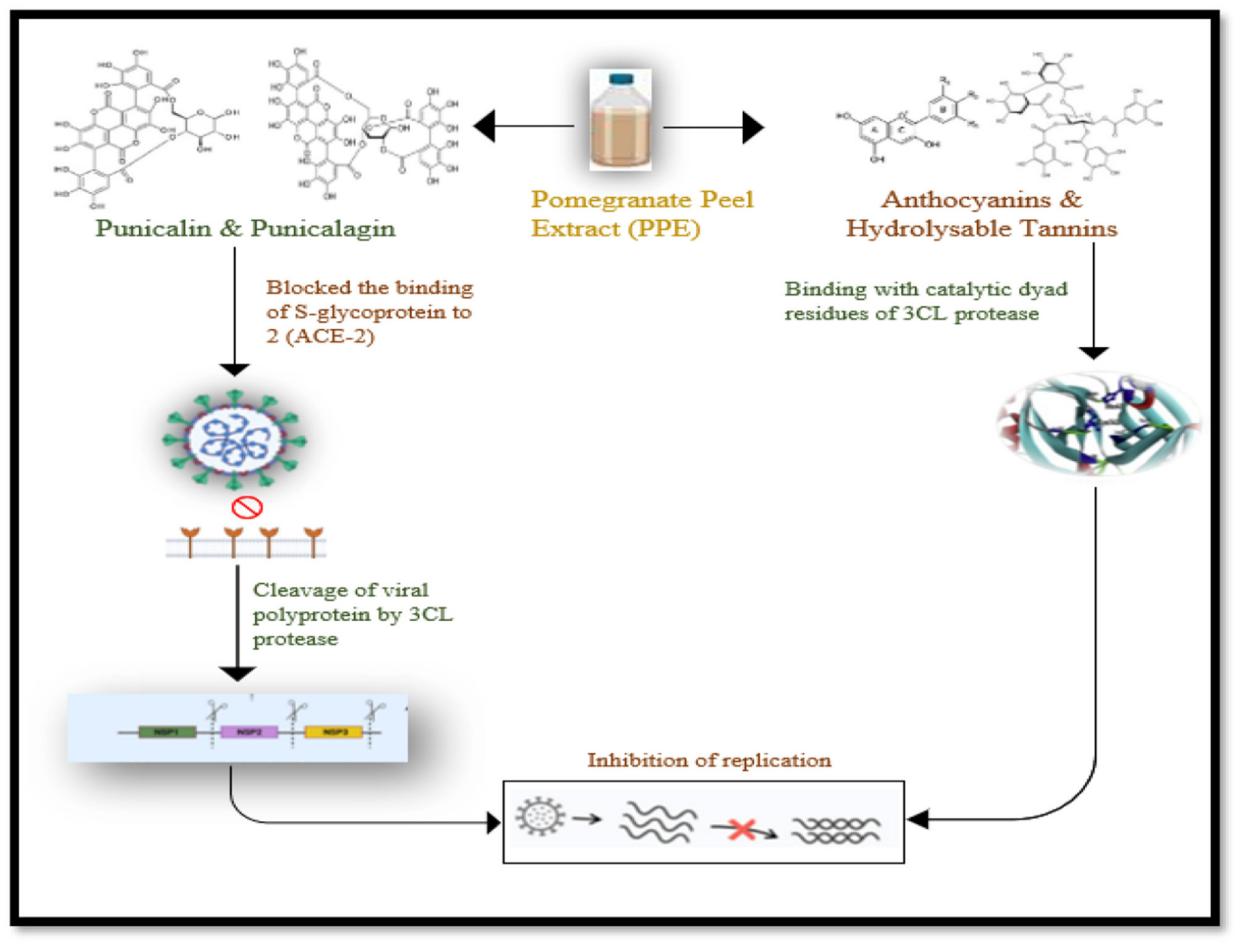
The anti-viral activity of punicalin and punicalagin and anthocyanins and hydrolysable tannins against viral replication of SARS-CoV-2.

Studies reveal that 50% of pomegranate's inedible portion contains bioactive compounds, including tannins, which serve as significant anti-viral agents due to their astringent capacity (44). Pomegranate waste, often overlooked in food processing, is a promising antiviral agent and a crucial product in pharmaceutical industries due to its effective chemical compounds, with studies showing its effectiveness against various viruses (45). The ethanolic pomegranate peel extract exhibits significant anti-viral activity against alphavirus, herpes, and influenza viruses by inhibiting viral replication and disrupting cellular machinery, and its bioactive compounds also have anti-viral properties (46). The pomegranate peel extract demonstrated significant anti-viral activity against influenza virus, inhibiting virus replication through hydrolysable tannins, gallagic acid, luteolin, and hydroxy-benzoic acid, with high concentrations (47). The anti-adinoviral activity of pomegranate peel extract evaluated by MTT essay. An important component in pomegranate peel extract, which is gallic acid, identified as potential anti-viral phytochemical component with highest concentration. Many other beneficial metabolites identified in PPE including ellagitannins, organic acid, anthocyanins and gallic acid which play significant role in inhibition of adenovirus replication, growth and adsorption (48). The pomegranate peel extract has shown therapeutic effects against various viral diseases, particularly SARS-CoV-2. Its anti-viral bioactivity has been significant against HIV in *in vitro* studies. The extract's phytochemical components, such as punicalagin and punicalin, block the binding of S-glycoprotein to ACE-2 receptors as shown in Figure 7.

### Anti-diabetic activity of pomegranate

Studies show pomegranate extracts and parts have therapeutic potential due to their diverse phytochemicals, including flavonoids, tannins, ellagic acid, and catechins. Food industries use by-products for functional foods (46, 50). The specific phytochemicals of pomegranate peel demonstrated significant interactions with α-amylase and α-glucosidases, helping to manage diabetes effectively by regulating glucose level due to their strong antidiabetic potential. These interaction contribute to proper glucose metabolism (51). The pomegranate seed oil (PSO) possesses potential antidiabetic activity due to abundance of major phytochemical such as punicic acid (PA) which involved in management of diabetes through various mechanisms including modulation of glucose homeostasis, reduction of oxidative stress and inflammatory cytokines. Pomegranate peel extract (PPE) also has anti-hyperglycemic effect for treatment of diabetes. The anti-diabetic activity of α-glucosidase showed significant results against diabetes by regulating glucose uptake. The findings of experimental study reported that the extract of pomegranate peel significantly lower the glucose level by effective anti-diabetic mechanism. The peel extract improved insulin secretion and glycemic control that lead to regulation of blood sugar level (51, 52). This study identified the high concentration of polyphenolic content including flavonoids and polyphenols in hydroalcoholic pomegranate leave extract. The maximum concentration of these polyphenols in pomegranate leave extract showed significant inhibitory effect against α-amylase, insulin sensitivity and glucose uptake and thus help to treat diabetes. The hydroalcoholic and aqueous of pomegranate leave extract exhibits potential anti-diabetic effect against complications associated with diabetes diseases (15). The study found that pomegranate extract, including its leaves, peels, and juice, effectively reduced fat accumulation, cholesterol, apoptosis, and inflammation in rats, and also reduced insulin resistance (53).

### Anti-hypertensive activity of pomegranate

Epidemiological studies highlight the significance of dietary modifications in reducing cardiovascular disease incidence. Among these, pomegranate leaf, peel, and seed contain bioactive components with cardioprotective properties, which are important for managing heart diseases, along medicinal treatment, lifestyle changes and therapeutic diet (54). The therapeutic benefits of compounds found in pomegranate peels and seeds, such as hydrolysable tannins, ellagitannins, and ellagic acid. These compounds help regulate blood pressure, reduce atherosclerosis, and lower the risk of hypertension and coronary and peripheral artery diseases (39). Use of supplementation of pomegranate peel extract for 8 weeks showed significant reduction in triglycerides level and lipid peroxidation (55). The pomegranate seeds enriched with tocotrienols, polyphenols, triterpene, and phospholipids, which exert antihypertensive activity by reducing blood pressure and other biomarkers (56, 57). A previous study showed the suppression of angiotensin-converting enzyme (ACE) enzyme to treat hypertension and pomegranate peel extract (58). Therapeutic effect of ellagic acid and proanthocyanidins in pomegranate peel extract showed significant results to reduce serum lipid profile and provide protection from risk of cardiovascular diseases (43).

### Anti-hepatotoxic activity of pomegranate

Pomegranate has the ability to scavenge excessive levels of reactive oxygen species (ROS) due to action mechanism of various phytochemical components with antioxidant properties. Polyphenolic compounds such as polyphenols, particularly ellagitannins, tannins and anthocyanins help to reduce oxidative stress and enhance the activity of liver enzymes including serum alanine aminotransferase (ALT) and aspartate aminotransferase (AST). These phenolic components also reduce toxicity by modulating the toxicity mechanism. Pomegranate peel extract have potential antioxidant and anti-hepatotoxic activity against oxidation of hepatic cells (59). Therapeutic potential of pomegranate seed oil (POS) has multitudinous effects against hepatotoxicity. These antioxidants and other phytochemical compounds possess anti-hepatotoxic activity against α-A induced poisoning rats by targeting oxidative stress-induced hepatocyte apoptosis. PSO contain number of anti-inflammatory compounds such as punicic acid and gallic acid against oxidative biomarkers. The study revealed that PSO has significant results against inflammatory damage in the liver (60). The study has discussed that pomegranate peel possesses ability to minimize the toxicity level of Carbon tetrachloride (CCl4) in liver. The pomegranate peel promotes the modification of inflammatory pathways The results of that study showed that 4.5% of pomegranate peel exert more antioxidant activity and improved liver function. The phenolic compounds of pomegranate peel have antihepatotoxic potential and enhance the functions of liver enzymes such as levels of aspartate transaminase (AST), alanine transaminase (ALT), and alkaline phosphatase (ALP) (61). The PPE also exert potential influence on liver fibrosis against CCl4 induced rat liver. The hepatotoxic damage significantly improved by application of PEE supplementation (62). This study evaluated the anti-hepatotoxic effects of pomegranate peel extract in rats with CCl4-induced hepatotoxicity. Extracts with concentrations ranging from 1 to 10,000 μg/mL significantly reduced liver toxicity, oxidative stress, and injury. Polyphenolic compounds like ellagic acid and ellagitannins played a key role in these effects. Both alcoholic and ethyl acetate extracts of pomegranate effectively inhibited lipid peroxidation, offering protection against hepatotoxicity (63). The pomegranate peel extract administrated with 200 mg/kg concentration, reduced significantly serum marker enzymes and improved liver enzymes activity by action of mechanism of phytochemical component including ellagitannin, flavonoids, phenolic acid and caffeic acids in PPE (64).

### Anti-osteoporosis activity of pomegranate

Pomegranate peel extract has potential anti-osteoporosis properties that help prevent the progression of bone loss. Several phytochemical compounds present in PPE such as anthocyanins, have the ability to protect from degenerative diseases including osteoporosis and osteopenia. A large number of polyphenolic components such as hydrolysable tannins, punicalagin, flavonoids and ellagic acid contribute to the therapeutic effects on bone mineral homeostasis markers (65). Pomegranate has the ability to reduce the bone loss, caused by postmenopausal osteoporosis and improve bones efficacy. A wide range of polyphenolic compounds present in pomegranate, one of them is punicalagin (PUN) that has ability to enhance bone resorption. The therapeutic potential of PUN has anti-inflammatory property by inhibiting pro-inflammatory osteoclast gene expression. The finding of the study confirm that pomegranate peel extract promote osteoblastic functions (39). The nutritional benefits of PPE promotes osteoblastic functions. The nutritional benefits of PPE make it particularly beneficial for management chronic diseases including osteoporosis and bone loss (66). The nutritional management of pomegranate seed oil extract (PSOE) has the ability to limit bone loss, osteoporosis and osteopenia. The therapeutic potential effect of PSOE help to treat bone microarchitecture impairment by enhancing antioxidative property of phytochemical components. The results showed significant reduction in progression of osteoporosis through PSOE. The study also showed that PPE has ability to reduce bone mineral density by inhibiting major key osteoclast markers. Polyphenols in PPE, such as ellagic acid and tannins contribute to improve bone formation and reducing inflammation. These phytochemical components of PPE promote the proliferation and activity of osteoblasts, helping to improve bone strength and mineral density by inhibiting osteoclast activity (66).

### Industrial uses of pomegranate by products

The by-products of pomegranate peel from industries managed sustainably through various approaches. Here are some strategies to promote economic circulation, address food insecurity, and enhance human health:

- Nutritional Supplements:

The pomegranate peel and other by-products of pomegranate are utilized in managing various nutritional requirements. Nutritional deficiencies and other health related diseases can be treated by nutritional supplementation with pomegranate peel. Pomegranate peel, as a nutraceutical and supplement has shown significant potential in attenuating various chronic health problems and fulfilling individual nutritional needs. The pomegranate peel enrich with natural additives that play an important role in nutraceutical industries (67). The pomegranate peel extract considered one of the best additive for enhancing food quality and taste, as it is enriched with natural food additives. The pomegranate peel extract (PEE) is encapsulated and used in various applications due to it's exceptional nutraceutical properties. The biological properties of PPE encapsulation help to fight against number of human related diseases (68). Muffin cakes made by pomegranate peel offer multiple functional food benefit and serve as an alternative to other types of cakes. The nutritional content of these pomegranate peel muffins contains enough amount of essential nutrients including calcium, dietary fiber, potassium, magnesium and other polyphenolic components which provide antioxidant properties. However, the amount of pomegranate peel added to the cake should not exceed 15%, as higher level may negatively impact the nutritional composition and overall formulation of muffin (69). Pomegranate peel extracts enhance antioxidant properties and reduce microbial contamination when used in fermented milk and cheese (70). Pomegranate peel, rich in natural additives and polyphenols, is used in developing functional and nutraceutical foods. Its infusion (PPI) significantly enhances antioxidant activity, lipid profiles, and feed efficiency in broiler chickens (71).

- Animal Feed:

Pomegranate peel contain numerous phytochemicals and polyphenolic compounds that protect against various cattle diseases and enhance cattle feed, due to presence of ellagitannin, an important antioxidant. The high antioxidant level in ellagitannin play major role in improving cattle health. It's exceptional properties help reduce oxidation and cholesterol levels in cattle. The potential use of pomegranate peel and its extracts in the nutraceutical industry support the development of cattle feed supplement (72). An experimental study showed that supplementing a 100 kg diet with 10 g of pomegranate peel improved chick growth and significantly increased their weight. Pomegranate peel extract also benefits other animals by enhancing growth, nutrient digestibility, and absorption (73). A study showed that supplementing cows with 4% pomegranate peel extract significantly improved digestibility, feed intake, and metabolism. Additionally, the extract enhanced the milk protein level in cows (74). Another study examined the effect of pomegranate peel extract on oxidative stress in dairy cows. The pomegranate peel (PP) supplemented with high fatty acids (FA) diet (15 g/kg) specifically polyunsaturated fatty acids and fed to dairy cows. The effect of PP supplementation and FA on metabolic profile and oxidative stress showed that it significantly improved lactation productivity, milk fatty acid composition and oxidative stress in dairy cows (75).

- Composting:

The pomegranate peel extract plays avital role in enhancement of plant growth, microbial protection and reduce need of fertilizers. The pomegranate peel compost on plants to identify the effect of PPE in replacement of other chemical fertilizers. The application of spray of pomegranate peel extract with showed significant improvement in soil fertility, plant growth, increased the process of photosynthetic pigments, enhance in amount of all essential minerals in soil and also increase the level of carotenoids in plant composition (76). A previous study showed that pomegranate peel with compost tea give significant antifungal on various harmful fungicides. Pomegranate peel showed effective results against soil borne pathogens by suppressing the activity of these fungicides on affected plants. The fungicidal treatment of pomegranate tea clearly showed that aqueous extract of pomegranate peel effectively manage the soil borne disease (77). The results of study revealed that nutrient-rich fortified pomegranate peel powder provide beneficial effects for plant growth and protection from harmful fertilizers. The sustainable management of plants using fortified pomegranate peel helps reduce the need for chemical fertilizers and acts as a beneficial biofertilizer. The application of pomegranate peel powder to soil enhances freshness and promotes the growth of various vegetative plants (78). Pomegranate peel powder contain large amount of natural additives, polyphenols and possesses the ability to remove ammonium ions from water and aid in nutrients adsorption. Sustainable management of pomegranate waste and by-products provide many benefits in the field of agriculture. A concentration of 400 mg of pomegranate peel powder contains functional groups, that effectively remove ammonia in water sample (79). The biological characteristics of pomegranate peel and its phytochemical components make it more valuable organic fertilizer for plants. The formulation of pomegranate peel powder and its ethanol extract on growth of sage herbs and the results showed significant growth of plant and enhance its chemical composition with essential nutrients (80).

- Natural Dyes:

The application of pomegranate peel plays significant role in textile industry due to presence of natural dyes in chemical composition. An aqueous extract of pomegranate peel demonstrates effective antiradical activity due to photoproduction of phenols and ellagic acid. Additionally, pomegranate peel and zinc oxide are incorporated into fabrics to protect against UV radiations. The wide range of organic photoprotective components, present in pomegranate peel and its extracts is extensively used in various dye techniques (81). The study evaluated the effect of pomegranate peel on different dyed substrate and identified its bio-functional activities. Pomegranate peel identify the polyamide fabrics effect against different microbes. The concentration of pomegranate peel, its extracts and natural dyes significantly enhanced the dye ability and antibacterial activity. Many extracts of pomegranate peel provide multiple protection from Gram-negative bacteria in the textile industry (49, 82). The application of pomegranate peel and its extracts proven as eco-friendly environment in textile industry. The solvent extraction of pomegranate peel effectively enhances the washing fastness activity and involve in shading the fabric with different colors without mordanting methods. The pomegranate peel possesses significant importance in dying process of fabric due to presence of non-toxic natural dyes (83). The results of experimental study showed that pomegranate peel extract possess potential importance in textile field. The potential dying properties of pomegranate peel involve the dying of silk fabric by dye fiber and natural mordants. It also the involvement of enhancement of fabric surfaces. The pomegranate peel under optimal parameters such time and temperature effect the quality and dying level in fabric (3). The study found that pomegranate peel and its extracts contain a large amount of phytoconstituents, which act as stabilizers in the textile industry. The polyphenolics in pomegranate peel can convert silver to its nano form, providing fabric stability. Additionally, the organic dye in pomegranate extract plays a key role in changing the color of fabric (84).

- Cosmetic Industry:

Pomegranate peel, seeds and seed oil are extracted using different extraction techniques such solvent extraction and supercritical fluid extraction. A large number of phytochemical components in pomegranate rind and seed including ellagic acid and punicalagin. These two phytochemicals play a significant role to enhance skin health by suppressing production melanin through tyrosinase. The action mechanism of ellagic acid in pomegranate extract help to lower the oxidative stress and increase anti-inflammatory properties in skin (9). The applications of pomegranate peel and its extract provide extensive advantages in cosmetic industries. Pomegranate peel and its extracts comprises various natural preservatives and bioactive components that play vital role in cosmetic formulation. The natural bioactive components mainly polyphenols provide potential protection from microbes in cosmetic products. The pomegranate peel extract with concentration of 10 mg/disc provide antimicrobial effect in cosmetic products by providing inhibited zone against microbial growth of *Aspergillus* (85). Pomegranate peel extract, rich in bioactive compounds, provides antioxidant and anti-aging benefits by protecting skin from UV A and UV B rays. Methanol and ethanol extracts show potential for sunscreen manufacturing, while the hydro-alcoholic extract, rich in polyphenols and flavonoids, helps scavenge harmful radicals and support skin health (86). Pomegranate peel and its extract various pharmacological properties which increase the productivity of better and safe quality cosmetic products. Results of previous study demonstrated that administration of pomegranate peel extract which comprises numerous phytochemical including punicalagin, gallic acids, ellagic acid-hex, anthocyanins increases the level of glutathione (GSH) and reduce the malondialdehyde (MDA) in the bloodstream and improve skin health (87). In this study, pomegranate peel extract showed potential role skin whitening by inhibiting an enzyme, tyrosinase. The pomegranate peel extract inhibited the tyrosinase activity in skin cells by tyrosinase hydrophobic binding pocket and reduce the activity of melanin producing enzyme. The phenolic concentration in extract help to give whitening effect and promote the collage production in the skin (88).

- Biogas Production:

The demand of energy production is increasing rapidly. Different sources of energy productions are natural gas, coal and other fossil fuels which use widely used for energy. Agricultural and industrial wastes are renewable energy sources and can be use in replacement of other energy sources. Pomegranate peel, a major industrial waste, possesses significant potential for energy production, particularly as fuel obtained through pyrolysis. The organic matter of pomegranate peel used in anaerobic digestion to produce biogas, contributing to renewable energy sources. The ash content of pomegranate peel obtained from thermal process comprise sufficient amount organic residues (89). The ash content of pomegranate peel comprises of many essential residues char, which contribute to play vital role as activated carbon. Through process of pyrolysis, the residues of pomegranate peel obtained biogas. By pyrolysis process, enough amount of organic matter:activated carbon, nitrogen and hydrogen obtain by using potassium hydroxide. The chemical activation during the process makes it time saver and faster than other biogas making methods. The residues of pomegranate peel use for renewable energy production as they provide activated carbon and maximum nitrogen adsorption. As the chemical composition of pomegranate peel represented that 46.48% carbon, 0.05% sulfur, and 3.13% ash material, the high absorbent material in pomegranate peel is less harmful after reducing content of ash, CO_2_, and other heavy metals (89). By adopting these strategies, industries can contribute to a circular economy, enhance human nutrition, and promote environmental sustainability.

## Conclusion

Pomegranate fruit (*Punica granatum* L.) is widely grown in Mediterranean regions due to its climate resilience. The pomegranate has been investigated for its biological properties due to the discovery of various bioactive chemicals, including ellagitannins such as punicalagin, punicalin, punicic acid, and ellagic acid. These substances have beneficial impacts on human health and have been evaluated *in vitro* and *in vivo* for their ability to battle diseases such as cancer, obesity, diabetes, influenza, bacterial infections, and inflammation. Nonetheless, it is critical to investigate the mechanisms of action and conduct *in vivo* metabolic assays to assess efficacy and side effects, as well as to determine appropriate concentrations for the use of current bioactive compounds in the cosmetic, pharmaceutical, and food industries. Further exploration of pomegranate peels, seeds and oil in the diet may offers effective strategy for treatment of various diseases.
